# Longitudinal measurement invariance of the meaning in life questionnaire in Chinese college students

**DOI:** 10.3389/fpsyg.2022.1001548

**Published:** 2022-10-25

**Authors:** Jie Luo, Fu-Chuan Tang, Ren Yang, Jie Gong, Cheng-Kui Yao, Xinquan Huang, Wei Chen, Shuo-Ying Zhao

**Affiliations:** ^1^School of Psychology, Guizhou Normal University, Guiyang, China; ^2^Office of Academic Research, University of Finance and Economics, Hangzhou, China; ^3^The School of Psychology and Cognitive Science, East China Normal University, Shanghai, China; ^4^School of Marxism, Guizhou Medical University, Guiyang, China; ^5^Department of Psychology, Kaili University, Kaili City, China

**Keywords:** longitudinal measurement invariance, meaning in life, presence of meaning in life, search for meaning in life, MLQ, Chinese college students

## Abstract

The Meaning in Life Questionnaire (MLQ) is a popular tool to measure the presence of and one’s search for meaning in life. Although the validity of the MLQ has been verified in previous studies, the evidence from longitudinal measurement invariance (LMI) of the MLQ is still lacking. The current study aimed to examine the LMI of the MLQ in a sample of Chinese college students (*N* = 328) at a 1-year interval. Multigroup confirmatory factor analysis (MCFA) was used to examine the LMI of the MLQ over four time points (over the course of 1 year). Results indicate that the MLQ has strict longitudinal invariance across 1-year in Chinese college students, and the latent means difference of MLQ-P is not significant differences across time, while the latent means difference of MLQ-S show significant differences between Time 1 and the other time points. Moreover, the internal consistency reliabilities (e.g., alpha and omega) of the MLQ scores were acceptable at all four time points, and the stability coefficients across time were moderate. These findings provide preliminary evidence that the MLQ has satisfactory longitudinal properties in Chinese college students.

## Introduction

Meaning in life refers to the sense made of and the significance one feels regarding the nature of one’s being and existence (Steger et al., 2006). As one of the most popular concepts in positive psychology, meaning in life plays an important role in the development of mental health and well-being (King et al., 2006; Ryff and Singer, 2010; Glaw et al., 2016). Previous studies have shown that a lack of meaning in life can lead to numerous mental health disorders, such as anxiety (Shiah et al., 2015), depression (Salami et al., 2022), and loneliness (King and Hicks, 2021; Macià et al., 2021), even increasing the risk of aggressive behavior or suicide (Wilchek-Aviad, 2015). Therefore, in the prevention of or intervention against these mental health disorders, it is crucial to be able to measure meaning in life accurately (Chen et al., 2020).

Several tools have been developed to assess meaning in life over the past 40 years (Brandstätter et al., 2012). Popular questionnaires include the Purpose in Life Test (PIL; Crumbaugh, 1969), the Life Regard Index (LRI, Battista and Almond, 1973), and the Meaning in Life Questionnaire (MLQ; Steger et al., 2006). Compared to the first two tools, the MLQ has unique advantages. For one, the factor structure of the PIL has rarely been verified in empirical studies, despite its development initially stemming from meaning therapy (Reker and Cousins, 1979; McGregor and Little, 1998). Furthermore, the items used in PIL and LRI can be seen as problematic in the measurement of meaning in life. For instance, the PIL and the LRI contain items such as, “With regard to suicide, I have thought of it seriously as a way out” and “I feel really good about my life.” Such statements can also be measured based on concepts other than meaning, such as emotion (*cf*. Steger et al., 2006). Finally, according to Frankl, 1963 theory, the core content of meaning in life is the seeking of meaning, but this has rarely been supported using existing questionnaires which measure meaning in life (Steger et al., 2006).

To address these gaps in knowledge, Steger et al. (2006) developed and validated the 10-item MLQ to measure meaning in life. The MLQ includes Presence and Search subscales, with the Presence subscale representing the presence of meaning or purpose in one’s life, and the Search subscale measuring one’s search for meaning in their life. According to Steger et al. (2006), presence of meaning can be assessed separately from search for meaning (Steger et al., 2006).

The MLQ has been used widely since its development, and many studies in different cultural contexts (i.e., Western and non-Western countries) have shown that the MLQ has satisfactory psychometric properties (e.g., internal consistency, retest reliability, structure validity; see Table 1 for details). For example, an Italian study in adults showed that the Italian version of the MLQ had good reliability (both αs >0.80) and the original two-factor model was supported (CFI = 0.99, GFI = 0.94, AGFI = 0.91, RMSEA = 0.059; Negri et al., 2020). Some studies have also replicated the original structure model in Chinese populations (e.g., Wang and Dai, 2008; Liu and Gan, 2010; Chen et al., 2015). Chen et al. (2015) revealed that the Chinese version of the MLQ had high structural validity when maintaining the two-factor structure, and showed that the same conclusion was supported through Rasch analysis. Studies have also been done in specific groups such as patients with life-threatening illnesses (Naghiyaee et al., 2020), displaced people (Chika et al., 2019), and those caring for chronically ill people (Chan, 2014). The factor structures obtained were all the same as those compiled by Steger et al. (2006), and the internal consistency coefficients were all above 0.8. Overall, prior studies support the proposed psychometric properties of the MLQ, but none of these studies have tested the longitudinal properties of the MLQ, specifically longitudinal measurement invariance (LMI) of MLQ scores over time.

**Table 1:** Summary of findings of psychometric properties of the MLQ in prior studies.

Authors	Sample	Country	Method	Alpha	Fit indices
Góngora and Grinhauz (2011)	128 adolescents, Age 13–18 *M*_age_ = 15.65, *SD* = 1.58	Argentina	CFA	P: 0.79 S: 0.78	CFI: 0.99 NFI: 0.91 RMSEA: 0.03
Góngora and Solano (2011)	707 adults *M*_age_ = 34.12, *SD* = 12.43	Argentina	CFA	P: 0.82 S: 0.88	CFI: 0.91 GFI: 0.92 NFI: 0.90 TLI: 0.88 RMSEA: 0.11
	180 adolescents *M*_age_ = 15.58, *SD* = 1.58	Argentina	CFA	P: 0.80 S: 0.81	CFI: 0.92 GFI: 0.91 NFI: 0.87 TLI: 0.89 RMSEA: 0.08
Kossakowska et al. (2013)	397 adolescents	Poland	CFA	P: 0.86 S: 0.72 T: 0.79	GFI: 0.901 AGFI: 0.84 RMSEA: 0.116
Akin and Taʂ (2015)	356 undergraduate students	Turkey	CFA	P: 0.88 S: 0.76	CFI: 0.97 GFI: 0.96 AGFI: 0.93 SRMR: 0.065 RMSEA: 0.065
Aquino et al. (2015)	414 participants *M*_age_ = 28.2, *SD* = 9.56	Brazil	CFA	P: 0.85 S: 0.89	CFI: 0.95 GFI: 0.94 AGFI: 0.90 RMSEA: 0.086
Damásio and Koller (2015)	3,020 adults *M*_age_ = 33.92, *SD* = 15.01	Brazil	EFA、CFA、MI		CFI: 0.94 TLI: 0.92 RMSEA: 0.118 SRMR: 0.129
Çelik and Gazioğlu (2015)	350 high school students	Turkey	CFA	P: 0.88 S: 0.93	CFI: 0.95 GFI: 0.93 RMSEA: 0.094 SRMR: 0.063
Demirdağ and Kalafat (2015)	322 teachers	Turkey	CFA	P: 0.81 S: 0.85	CFI: 0.95 GFI: 0.93 AGFI: 0.89 RFI: 0.92 NFI: 0.93 RMSEA: 0.068
Singh et al. (2016)	826 adults aged 18–60 years *M*_age_ = 29.44, *SD* = 12.82	India	CFA	P: 0.78 S: 0.81	CFI: 0.94 GFI: 0.94 NFI: 0.93 RMSEA: 0.084
Wang et al. (2016)	5,510 students *M*_age_ = 16.77, *SD* = 3.268	China	CFA、MI	*p* > 0.78 S > 0.78	CFI: 0.926 TLI: 0.912 NFI: 0.930 RMSEA: 0.092 SRMR: 0.073
Rose et al. (2017)	135 adolescents aged 12–18 years, *M*_age_ = 15.18, *SD* = 1.42	Australia	CFA	P: 0.82 S: 0.84	CFI: 0.92 SRMR: 0.10
Stalikas et al. (2018)	1,561 adults *M*_age_ = 39.7, *SD* = 12.81	Greece	Bifactor、MI CFA、ESEM	P: 0.85 S: 0.86 T: 0.76	CFI: 0.978 TLI: 0.961 RMSEA: 0.052 SRMR: 0.040
Kolesovs (2019)	406 adults aged 18–49 *M*_age_ = 23.2, *SD* = 5.83	Latvia	CFA	P: 0.88 S: 0.84	CFI: 0.92 TLI: 0.89 RMSEA: 0.09 SRMR: 0.10
Satoshi and Kohki (2019)	2,000 adults over the age of 20	Japan	CFA、MI	20–24 years P: 0.90 S: 0.89	CFI: 0.922 RMSEA: 0.057 SRMR: 0.08 (Fixed project residual load)
				25–44 years P: 0.87 S: 0.88	CFI: 0.921 RMSEA: 0.06 SRMR: 0.081
				45–64 years P: 0.89 S: 0.91	CFI: 0.895 RMSEA: 0.06 SRMR: 0.085
				Over 65 years old P: 0.89 S: 0.90	CFI: 0.895 RMSEA: 0.06 SRMR: 0.103
Balgiu (2020)	329 college students *M*_age_ = 19.29, *SD* = 1.42	Romania	CFA、MI	P: 0.79 S: 0.85	CFI: 0.957 IFI: 0.958 TLI: 0.940 RMSEA: 0.073 SRMR: 0.071
Naghiyaee et al. (2020)	301 patients diagnosed with life-threatening diseases	Tehran	CFA、MI	P: 0.84 S: 0.88 T: 0.90	CFI: 0.99 GFI: 0.93 AGFI: 0.85 NNFI: 0.99 RMSEA: 0.065 SRMR: 0.05
Negri et al. (2020)	464 adults aged 20–60 years, *M*_age_ = 39.34, *SD* = 10.86	Italy	CFA	P: 0.84 S: 0.90	CFI: 0.99 GFI: 0.94 AGFI: 0.91 RMSEA: 0.059 SRMR: 0.064
Vela et al. (2020)	202 students, *M*_age_ = 22.42, *SD* = 6.22	Hispanic/Non-Hispanic (Spanish)	CFA	P: 0.66 S: 0.87 T: 0.77	CFI: 0.95 GFI: 0.92 TLI: 0.93 RMSEA: 0.08 SRMR: 0.08
Datu and Yuen (2022)	1,089 middle school students *M*_age_ = 14.88, *SD* = 0.99	Hong Kong, China	CFA	P: 0.84 S: 0.88	CFI: 0.931 GFI: 0.924 TLI: 0.905 RMSEA: 0.10

T, total scale; EFA, exploratory factor analysis; CFA, confirmatory factor analysis; Bifactor, the bifactor method of factor analysis; ESEM, exploratory structural equation model; CFI, comparative fit index; TLI, Tucker-Lewis index; NFI, normed fit index; GFI, goodness-of-fit index; AGFI, adjusted goodness-of-fit index; NNFI, non-normed fit index; IFI, incremental fit index; RMSEA, root mean square error of approximation; SRMR, standardized root mean square residual; P, presence subscales; S, search subscales.

### Measurement invariance of the MLQ

Measurement invariance (MI) is vital for cross-group comparison because the interpretation of mean differences across groups may be misguided and questionable unless latent constructs in different subgroups are equivalent (Byrne and Watkins, 2003; Chen, 2008). In other words, the establishment of MI is the precondition for cross-group comparison (e.g., male vs. female; Chen, 2008). Prior studies have tested the MI of the MLQ in various countries including Brazil, China, Greek, Japan, and Romania (e.g., Damásio and Koller, 2015; Stalikas et al., 2018; Satoshi and Kohki, 2019; Balgiu, 2020; Datu and Yuen, 2022). More specifically, the Brazilian version of MLQ showed strict invariance for gender and age groups (i.e., youth, adults, and the elderly) (Damásio and Koller, 2015). Likewise, gender invariance was verified in a Greek (Stalikas et al., 2018) and Romanian samples (Balgiu, 2020). The MI among Chinese students aged 10–25 has been verified and shows strict MI across age groups (i.e., early adolescence, middle adolescence, late adolescence, and early adulthood; Wang et al., 2016).

Measurement invariance involves cross-sectional data as well as longitudinal data, with the latter representing MI over different points in time. Although previous studies have focused on the MI of the MLQ across different groups (e.g., gender and age) through cross-sectional investigation, the LMI of MLQ scores has not been explored. Similar to MI, LMI also tests the equality of a construct for a tool, but its focus is on equality across time, not across groups (Millsap and Cham, 2012). LMI is a desirable equality, as its results can show that the same construct can be tested across occasions (i.e., configural, metric, scalar, and strict invariance), providing a necessary and stable foundation for the comparison of mean values in longitudinal research. According to Steger and Kashdan (2007), moderate stability was found for the two subscales of the MLQ across a longer term (i.e., over a 13-month period), with a stability coefficient of 0.41 for the Presence subscale (MLQ-P) and 0.50 for the Search subscale (MLQ-S). Moreover, prior longitudinal studies have tested the correlation between meaning in life and other covariates in health and positive psychology (e.g., Hsiao et al., 2013; Negru-Subtirica et al., 2016; Park et al., 2020; Shek et al., 2021), yet these studies did not examine whether meaning in life and its two components have MI over time. The present study is thus the first to measure whether the MLQ has LMI across time.

### The current study

The main purpose of the present study was to examine the LMI of MLQ in a sample of Chinese college students. Confirmatory factor analysis (CFA) was conducted to test whether the MLQ scores had LMI across time, testing the configural, metric, scalar, and strict invariance over a 12-month interval. Based on Steger et al. (2006)’s original work, we assumed that the LMI of MLQ could be verified. Finally, the internal consistency indices (i.e., Cronbach’s α, McDonald’s ω, and mean inter-item correlation), stability coefficients, and latent factor means comparison were also tested.

## Materials and methods

### Participants

The sample for the present study was drawn from a normal university in Guizhou province, China. A Monte Carlo study suggested that a sample size of 200 would be needed for a power of 0.80 in a CFA model (Muthén and Muthén, 2002), and sample would be used to test the longitudinal properties of the MLQ across a 1-year interval with four waves. A total of 328 college students comprising 50 males (15.2%) and 278 females (84.8%) took part in the first survey in April 2018 (Time 1; aged 18 to 24; *M*_age_ = 20.82, *SD* = 1.18). The second survey involved the same participants as in August 2018, and took place at an interval of 3 months (Time 2). The third and fourth surveys were conducted in December 2018 (Time 3) and April 2019 (Time 4), respectively, with no sample loss. The sample was made up of 153 sophomores (46.6%) and 175 juniors (53.4%). In terms of cultural background, 197 students (60.1%) were of Han ethnicity, with the remaining 39.9% of ethnic minority backgrounds (*N* = 131). Finally, 150 students majored in science (45.7%) and 178 students in liberal arts (54.3%).

### Procedure

The study questionnaire was completed in a classroom setting while participants attended their classes. More specifically, the investigation was announced to the students in class by the lecturers (who included the researchers), which had been agreed upon earlier in discussion with the researchers of the current study. Participants completed the paper and pencil questionnaire and returned it to the study administrator directly after completion. To facilitate identification and follow-up, each participant was given a random number at the baseline survey point, and participants completed the questionnaire at four times over the 1-year period of the study. Before beginning the formal study, all participants were informed as to the nature, goal, confidentiality, and anonymity of the study, and were told that their responses would not affect their school performance. The full investigation followed the principle of voluntariness and participants were able to withdraw from the study at any time. This study questionnaire took about 5 to 8 min to complete each time. The study was approved by the Subjects Review Committee of Guizhou Normal University (GZNUPSY.No2018M[005]).

## Measures

### The meaning in life questionnaire

The MLQ is a self-report questionnaire which was developed to assess one’s attitudes regarding the presence of and search for meaning in life (Steger et al., 2006). It includes 10 items and two factors: Presence (MLQ-P), and Search (MLQ-S). Five items assess the presence factor (e.g., “My life has a clear sense of purpose”) and five items assess the search factor (e.g., “I am looking for something that makes my life feel meaningful”). Each item is rated on a seven-point scale ranging from 1 (absolutely untrue) to 7 (absolutely true). The higher the MLQ scores, the higher the happiness experienced (Steger et al., 2009). The Chinese version of the MLQ (C-MLQ) has been validated and showed adequate internal consistency, and good factorial validity and construct validity (Wang and Dai, 2008). In the present study, the alphas for MLQ-P and MLQ-S at the four time points were 0.78/0.81, 0.84/0.85, 0.85/0.86, and 0.85/0.84, respectively.

### Data analysis

First, descriptive statistics (e.g., *M*, *SD*, *SK*, and *KU*) for the MLQ were performed using SPSS 26.0 (IBM Crop, 2019). Then, a series of CFAs were performed using Mplus 7.0 (Muthén and Muthén, 1998) to examine the LMI across the four time points (over a 12-month period). Given that the values of the skewness and kurtosis in certain items were not within a range of −1 to +1 (e.g., items 1, 2, 8, 9, and 10; see Table 2), the maximum likelihood estimation with a mean-adjusted chi-square (MLM) was robust. The model fits of the CFA were also calculated, including the comparative fit index (CFI), the Tucker-Lewis index (TLI), and the root mean square error of approximation (RMSEA). If the values of CFI and TLI are both higher than 0.90 and RMSEA is lower than 0.08, it indicates an acceptable model fit. If CFI and TLI are above 0.95 and RMSEA is below 0.05, it indicates good model fit (Hu and Bentler, 1999; Kline, 2010).

**Table 2:** Descriptive statistics for the MLQ scores over time.

Item	Time 1			Time 2					Time 3			Time 4				
	*M (SD)*	*SK*	*KU*	*CITC*	*M (SD)*	*SK*	*KU*	*CITC*	*M (SD)*	*SK*	*KU*	*CITC*	*M (SD)*	*SK*	*KU*	*CITC*
1	5.25 (1.19)	−0.62	0.04	0.55	5.33 (1.20)	−0.91	0.98	0.64	5.28 (1.22)	−1.09	1.76	0.65	5.22 (1.21)	−0.96	1.35	0.67
2	5.88 (1.10)	−1.36	2.72	0.45	5.76 (1.06)	−1.32	2.61	0.48	5.67 (1.18)	−1.35	2.62	0.59	5.39 (1.24)	−1.05	1.43	0.51
3	5.16 (1.52)	−0.79	−0.03	0.60	4.84 (1.54)	−0.60	−0.37	0.66	4.76 (1.51)	−0.34	−0.57	0.68	4.55 (1.52)	−0.43	−0.40	0.72
4	4.84 (1.34)	−0.42	−0.08	0.63	4.86 (1.27)	−0.31	−0.22	0.76	4.87 (1.35)	−0.37	−0.39	0.74	5.06 (1.26)	−0.66	0.50	0.76
5	5.09 (1.31)	−0.40	−0.20	0.59	5.13 (1.29)	−0.59	0.22	0.67	5.11 (1.33)	−0.87	0.87	0.68	5.15 (1.28)	−0.81	0.61	0.69
6	4.59 (1.48)	−0.31	−0.41	0.56	4.61 (1.40)	−0.25	−0.26	0.69	4.60 (1.41)	−0.34	−0.26	0.70	4.64 (1.39)	−0.16	−0.26	0.72
7	5.08 (1.47)	−0.76	0.20	0.68	4.83 (1.47)	−0.61	0.01	0.73	4.68 (1.34)	−0.46	0.21	0.74	4.52 (1.49)	−0.37	−0.35	0.72
8	5.38 (1.47)	−1.05	0.72	0.62	5.26 (1.41)	−0.99	0.66	0.73	5.23 (1.38)	−0.91	0.70	0.71	5.01 (1.40)	−0.84	0.46	0.65
9	4.30 (1.65)	−0.09	−0.80	0.47	4.41 (1.71)	−0.06	−1.01	0.55	4.45 (1.63)	−0.28	−0.73	0.59	4.41 (1.62)	−0.07	−0.81	0.53
10	5.36 (1.37)	−1.15	1.38	0.61	5.20 (1.38)	−1.06	0.93	0.70	5.09 (1.44)	−0.98	0.64	0.71	5.03 (1.37)	−0.83	0.40	0.62

SK, skewness; KU, kurtosis; CITC, corrected item-total correlations with each item’s respective factor.

Second, the LMI of the MLQ was examined by qualifying the model parameters equality between a series of nested models (i.e., configural, metric, scalar, and strict invariance models). The configural invariance model hypothesis states that the factor structure should follow the same pattern over all time points (Millsap and Cham, 2012). In the second step, the metric invariance model required that the corresponding factor loadings were equal across all time points. Then, in the scalar invariance model, the item intercepts would also be set as equal on the basis of the previous model. Finally, the strict invariance model set the corresponding factor loadings, intercepts, and error variances to be equal across time points (Wang et al., 2012). LMI is achieved (configural, metric, scalar, and strict invariance) when the difference between the unconstrained and constrained models are not significant. Because the chi-square difference test is sensitive to sample size, we examined the changes in CFI, RMSEA, and SRMR to compare the nested models (Chen, 2007). As recommended by Chen (2007), to test the metric invariance model, changes in CFI (ΔCFI) of ≥0.01, supplemented by changes in RMSEA (ΔRMSEA) of ≥0.015 and changes in SRMR (ΔSRMR) of ≥0.03 indicates an absence of MI. When testing the scalar and strict invariance model, ΔCFI of ≥0.01, supplemented by ΔRMSEA of ≥0.015 and ΔSRMR of ≥0.01 indicates an absence of MI.

Third, the internal consistency coefficients of the MLQ at each time point were calculated, including Cronbach’s α, McDonald’s ω, and mean inter-item correlation (MIC). According to Barker et al. (1994), a Cronbach’s α of 0.70 to 0.79 = acceptable, 0.80 to 0.89 = good, and above 0.90 = excellent. A McDonald’s ω greater than 0.70 is acceptable (Hair et al., 2014). Finally, if the MIC value ranges from 0.15 to 0.50 the correlation coefficients should be acceptable (Clark and Watson, 1995). In addition, the stability coefficients were also obtained by testing the correlations between the factors while the intraclass correlation coefficient (ICC) was also calculated for all four time points.

Finally, we explored the differences in the MLQ at the different time points by comparing the latent factor means across time based on LMI. Specifically, the MLQ two-factor means were set at Time 1 to zero, and while the latent factor mean was estimated freely at the other time points (i.e., Times 2, 3, and 4).

## Results

### Descriptive statistics

Descriptive statistics results for the MLQ scores at the four time points are shown in Table 2, including the mean, standard deviation, skewness, kurtosis, and corrected item-total correlations with each item’s respective factor (CITC).

### Factor structure and LMI of the MLQ

As shown in Table 3, the model indices of the MLQ at the four time points achieved acceptable levels (i.e., CFIs and TLIs >0.90, RMSEAs <0.08), with the exception of the TLI at Time 1, which was 0.894. However, this was also suitable for subsequent analysis based on the CFI and RMSEA. A series of nest models were then used to compared the LMI of the MLQ across the time points (i.e., the configural invariance model, the metric invariance model, the scalar invariance model, and the strict invariance model; see Table 3).

**Table 3:** Longitudinal measurement invariance model fit statistics for the MLQ.

Model	χ^2^	*df*	CFI	TLI	SRMR	RMSEA	Δχ^2^ (*p*)	ΔCFI	ΔTLI	ΔSRMR	ΔRMSEA
Time 1	123.060	34	0.920	0.894	0.083	0.071					
Time 2	114.622	34	0.946	0.929	0.073	0.071					
Time 3	122.950	34	0.945	0.927	0.068	0.072					
Time 4	134.681	34	0.945	0.927	0.075	0.068					
Configural variance	1180.230	652	0.937	0.925	0.067	0.041	–	–	–	–	–
Metric variance	1212.240	676	0.936	0.927	0.070	0.040	32.009 (0.127)	−0.001	0.002	0.003	−0.001
Scalar variance	1253.880	700	0.934	0.926	0.070	0.041	41.641 (0.014)	−0.002	−0.001	0.000	0.001
Strict variance	1364.360	730	0.924	0.919	0.072	0.043	110.474 (< 0.001)	−0.010	−0.007	0.002	0.002

df, degrees of freedom; CFI, comparative fit index; TLI, Tucker-Lewis index; SRMR, standardized root mean square residual; RMSEA, root mean square error of approximation; 90% CI, 90% confidence interval around RMSEA; Δχ^2^, change in Δχ^2^relative to the preceding model; p, p value of Δχ^2^; ΔCFI, change in comparative fit index relative to the preceding model; ΔTLI, change in Tucker-Lewis index relative to the preceding model; ΔSRMR, change in standardized root mean square residual to the preceding model; ΔRMSEA, change in root mean square error of approximation relative to the preceding model.

**Figure 1:**
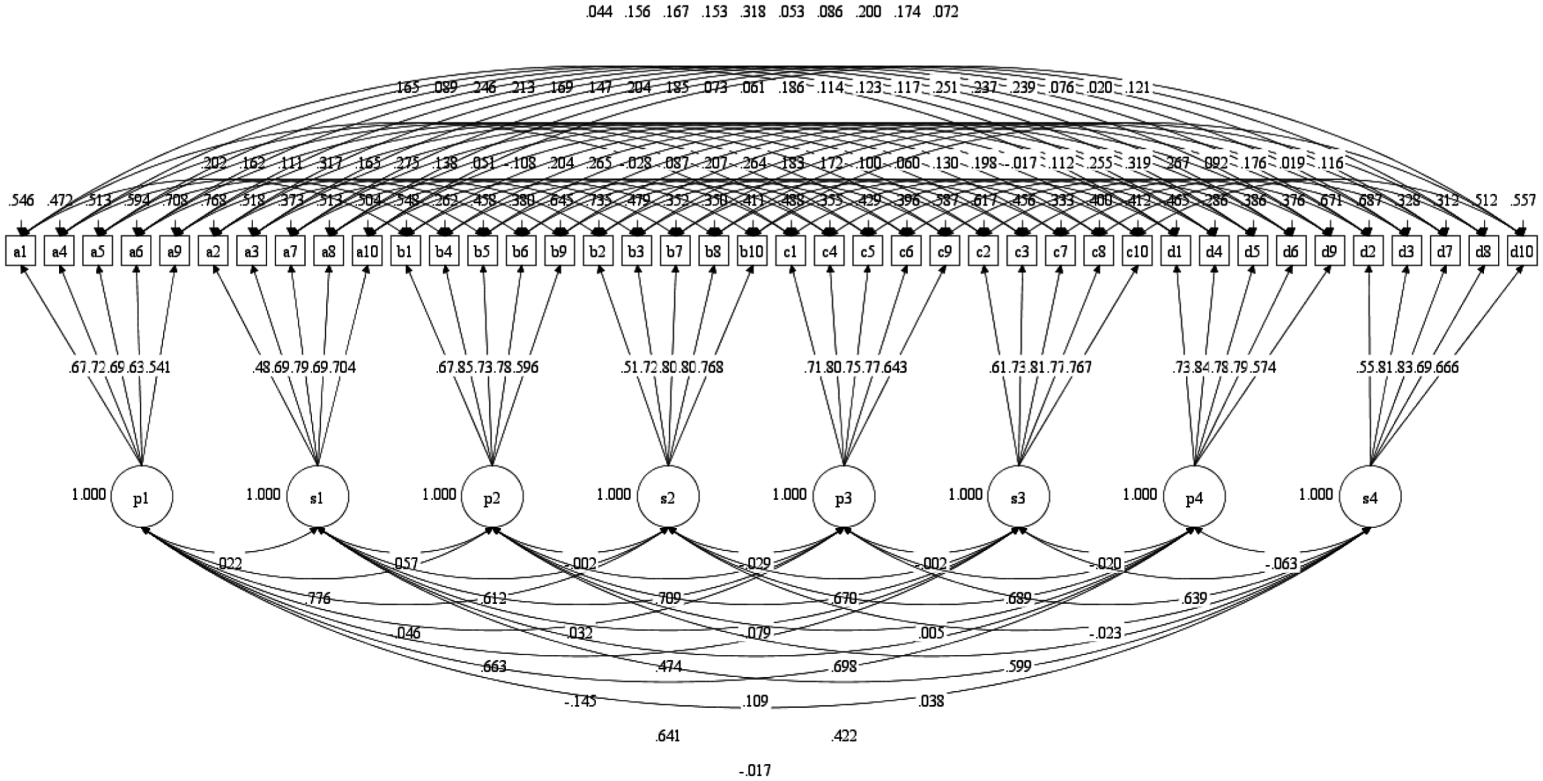
Diagram for the longitudinal configural invariance model. P1, presence of MLQ at Time 1; S1, search of MLQ at Time 1; P2, presence of MLQ at Time 2; S2, search of MLQ at Time 2; P3, presence of MLQ at Time 3; S3, search of MLQ at Time 3; P4, presence of MLQ at Time 4; S4, search of MLQ at Time 4.

First, the configural model had adequate model fit (CFI = 0.937, TLI = 0.925, RMSEA = 0.041). The correlations within and between factors for the model can be found in Figure 1.

The metric model fit was also satisfactory (CFI = 0.936, TLI = 0.927, RMSEA = 0.040). There were inappreciable differences in CFI, TLI, SRMR, and RMSEA between the configural and metric model (ΔCFI = −0.001, ΔTLI = 0.002, ΔSRMR = 0.003, ΔRMSEA = −0.001). These findings supported the metric invariance of the MLQ scores across time points. The fit indices of the scalar invariance model (CFI = 0.934, TLI = 0.926, RMSEA = 0.041) were also acceptable. Negligible changes (ΔCFI = −0.002, ΔTLI = −0.001, ΔSRMR = 0.000, ΔRMSEA = 0.001) indicated that scalar invariance was obtained.

Finally, strict invariance (CFI = 0.924, TLI = 0.919, RMSEA = 0.043) was also supported in terms of the change values of CFI, TLI, SRMR, and RMSEA (ΔCFI = −0.010, ΔTLI = −0.007, ΔSRMR = 0.002, ΔRMSEA = 0.002). In summary, our results demonstrated that the two-factor structure of the MLQ scores had LMI over the 1-year interval. The standardized factor loadings of the longitudinal factor solution are presented in Table 4.

**Table 4:** Standardized factor loadings for the longitudinal factor model of the MLQ.

Item	MLQ-P1	MLQ-S1	MLQ-P2	MLQ-S2	MLQ-P3	MLQ-S3	MLQ-P4	MLQ-S4
Item 1	0.678		0.699		0.710		0.701	
Item 4	0.790		0.806		0.816		0.808	
Item 5	0.726		0.745		0.756		0.747	
Item 6	0.729		0.748		0.759		0.750	
Item 9	0.574		0.595		0.608		0.598	
Item 2		0.550		0.563		0.558		0.564
Item 3		0.738		0.750		0.745		0.751
Item 7		0.802		0.812		0.808		0.813
Item 8		0.730		0.742		0.737		0.743
Item 10		0.712		0.724		0.720		0.726

MLQ, the Meaning in Life Questionnaire; MLQ-P1, presence of meaning in life at Time 1; MLQ-S1, search for meaning in life at Time 1; MLQ-P2, presence of meaning in life at Time 2; MLQ-S2, search for meaning in life at Time 2; MLQ-P3, presence of meaning in life at Time 3; MLQ-S3, search for meaning in life at Time 3; MLQ-P4, presence of meaning in life at Time 4; MLQ-S4, search for meaning in life at Time 4. All factor loadings are significant at the level of *p* < 0.001.

### Internal consistency, stability coefficients, and latent factor means comparison over time

The coefficient αs for the MLQ two-factor scores were acceptable (αs > 0.70) at all four time points, separately. As to the MLQ-P factor, the Cronbach’s αs were 0.78 at Time 1 (MIC = 0.43), 0.84 at Time 2 (MIC = 0.54), 0.85 at Time 3 (MIC = 0.55), and 0.85 at Time 4 (MIC = 0.55). The Cronbach’s αs at the four time points were 0.81 (MIC = 0.45), 0.85 (MIC = 0.52), 0.86 (MIC = 0.56), and 0.84 (MIC = 0.51) for the MLQ-S subscale, respectively. The McDonald’s ω results showed that the MLQ scores also had satisfactory internal consistency values. More specifically, for the MLQ-P subscale, the ωs were 0.79, 0.86, 0.86, and 0.86 at the four time points, respectively; for the MLQ-S subscale, the ωs were all above 0.80 (i.e., 0.81 at Time 1, 0.85 at Time 2, 0.86 at Time 3, and 0.84 at Time 4). Moreover, the results of the stability coefficients between Time 1, Time 2, Time 3, and Time 4 ranged from 0.64 to 0.76 for MLQ-P, and 0.43 to 0.67 for MLQ-S (*ps* < 0.001). The results of the intraclass correlation coefficient (ICC) were 0.62 for MLQ-P and 0.51 for MLQ-S (*ps* < 0.001). Finally, using the strict longitudinal invariance model, the mean of each factor at each of the four different time points could be compared meaningfully. More specifically, the latent mean was not significantly different between Time 1 and Time 2 (mean difference = 0.043, *p* = 0.344), Time 1 and Time 3 (mean difference = 0.038, *p* = 0.442), and Time 1 and Time 4 (mean difference = 0.089, *p* = 0.088) for MLQ-P. For the MLQ-S subscale, taking Time 1 as a reference, Time 2, Time 3, and Time 4 had significant differences in comparison to their latent means (i.e., mean difference = −0.194/−0.289/−0.453, *p*s < 0.001).

## Discussion

The purpose of this study was to examine the longitudinal properties of the MLQ (Steger et al., 2006), which was developed to assess the presence of and search for meaning in life, in a sample of Chinese college students. The results showed that the MLQ and its two subscales have strict longitudinal invariance over a 12-month interval (i.e., equality of factor patterns, factor loadings, item intercepts, and item uniqueness for all 10 items across a 1-year interval). Meanwhile, the internal consistencies and stability coefficients also supported the MLQ scores across time. Overall, our findings replicate and extend the findings of previous studies on the psychometric properties of the MLQ and, for the first time, validate the longitudinal properties of the MLQ across time.

### Longitudinal measurement invariance of the MLQ

Longitudinal measurement invariance was used to evaluate whether the same factor structure could be verified at different points in time (Wu, 2017). This is essential because the structure of meaning of life could develop or change as the time goes by (Steger et al., 2009). In other words, it is important that changes over time can be attributed to actual differences or individual developments (Dimitrov, 2010; Millsap and Cham, 2012). The aim of the present study was therefore to examine the LMI of the MLQ two-factor model. The results of this study showed that the MLQ had strict MI across time, which extends previous findings that measured invariance across gender and age groups (e.g., Damásio and Koller, 2015; Wang et al., 2016). While prior research has focused on MI across groups, the present study focused on MI across different time points. Our results indicated that meaning in life in Chinese college students had strict LMI over time (i.e., configural, metric, scalar, and strict invariance), at least in individuals aged 18 to 24 over an interval of a year. Consistent with Steger et al. (2009), meaning in life was shown to be stable over the course of a year, and was independent of other forms of happiness (Steger, 2009). This suggests that the MLQ was able to measure the same structure at the different time points, and that the two-factor structure in this study was good, consistent with previous studies. Importantly, this implies that the mean difference in meaning in life scores as measured through the MLQ over time can be considered as true changes in an individual’s meaning in life. However, given that the MI across age was inconsistent, future examinations of LMI of the MLQ should test the presented findings against other age group populations (e.g., younger adolescents or older adults).

### Internal consistency, stable coefficients, and latent factor means over time

The internal consistency coefficients also offered some meaningful information about the stability of MLQ scoring over time. Similar to previous findings (e.g., Negri et al., 2020), the coefficient αs of the MLQ factor scores were acceptable over time. Furthermore, the McDonald’s ω and MIC values also suggest that the MLQ has satisfactory and acceptable internal consistency across time.

As to the stability coefficient, the MLQ latent factor scores across the four time points were significantly correlated (i.e., *r*s ranged from 0.43 to 0.76). Moreover, the ICC indices showed that the stability coefficient for the MLQ subscale scores were moderate (Weir, 2005). Consistent with manifest factor correlations (Steger and Kashdan, 2007), the moderate to strong correlation between the latent factors as well as the ICC values suggested that meaning in life is at least moderately stable over a 1-year period (Steger and Kashdan, 2007).

As the LMI results of the MLQ were verified, the differences of the latent factor means could be compared for further exploration. Using Time 1 as a baseline for comparison, the latent means difference of the MLQ-P at Time 2, Time 3, and Time 4 were not significant (*p*s > 0.05). In contrast, the latent means difference of the MLQ-S at Time 2, Time 3, and Time 4 showed significant differences (*p*s < 0.001) when compared with Time 1. These results are contrary to the findings of Hsiao et al. (2013), who found significant differences in MLQ-P mean values over a 14-month follow-up period, but no differences in MLQ-S, even though meaning in life was not the primary variable of concern in the study. We speculate that one reason for these opposite results could be due to the differences in sample sampling. In our study, the samples comprised college students, while in the Hsiao study, the subjects were breast cancer survivors. Changes in the MLQ-S could also have been caused by different cognitive styles. Steger et al. (2008) showed that their findings supported with Maddi’s 1970 theory that search for meaning was associated with different cognitive styles, which were characterized by a tendency to question the status quo and continuous negative thinking about the past and present. Therefore, people may perceive reality in different ways over time intervals of more than a year, which may have implications on the search for meaning in life.

### Limitations and future directions

The findings from the present study must be considered with consideration of several limitations. First, the participants in the current study came from Southwest China, which may limit the generalizability of our results. Future research should consider including university students from other regions when attempting to replicate the current findings. Second, as the sample size and gender ratio was too broad, the current study did not measure the LMI of the MLQ in different gender or age populations. Future studies should extend to include such an exploration when measuring LMI in the context of balancing gender and population proportions from the various age groups. Third, we only examined the LMI of the MLQ scores at 3-month intervals between data collection; future studies should test the LMI of the MLQ over longer time intervals. Finally, the current study examined the LMI of the MLQ in college students (i.e., emerging adulthood). Future research should test the LMI of the MLQ in other demographics (e.g., community adults or younger adolescents).

Despite these limitations, the present study expands our understanding of the longitudinal properties of MLQ scores. Overall, the current study proves that the MLQ can be an effective measure to assess meaning in life of Chinese college students across time. Specifically, the MLQ was shown to have strict LMI (including configural invariance, metric invariance, scalar invariance, and strict invariance) across a 1-year interval. Future study should further explore this property of the MLQ.

## Data availability statement

The data that support the findings of this study are available from the first author, JL, upon reasonable request.

## Ethics statement

The studies involving human participants were reviewed and approved by the Subjects Review Committee at Guizhou Normal University. The patients/participants provided their written informed consent to participate in this study.

## Author contributions

JL was mainly responsible for the conception and design of this study, investigated and analyzed the data, drafted the manuscript, and provided the final approval for the manuscript. F-CT contributed to the analysis and interpretation of the data, drafted the manuscript, and provided the final approval for the manuscript. RY helped revise the manuscript and provided edits for the revision. JG and C-KY helped revise the manuscript. XH helped guide the revision and provided financial support. WC and S-YZ revised the manuscript, provided financial support, and provided the final approval for the manuscript. All authors contributed to the article and approved the submitted version.

## Funding

This study was funded by the Guizhou Philosophy and Social Science Planning General Project (18GZYB57), Guizhou Philosophy and Social Science Planning Key Project (21GZZD51), and Guizhou Province Science and Technology Foundation (Qian Ke He Jichu-ZK [2022] General 303).

## Conflict of interest

The authors declare that this research was conducted in the absence of any commercial or financial relationships that could be construed as potential conflicts of interest.

## Publisher’s note

All claims expressed in this article are solely those of the authors and do not necessarily represent those of their affiliated organizations, or those of the publisher, the editors and the reviewers. Any product that may be evaluated in this article, or claim that may be made by its manufacturer, is not guaranteed or endorsed by the publisher.
